# Bis[*N*-(2-hy­droxy­benz­yl)adamantan-1-aminium] fluoride tetra­fluoro­borate monohydrate

**DOI:** 10.1107/S1600536812000694

**Published:** 2012-01-14

**Authors:** Ying-Chun Wang

**Affiliations:** a College of Chemistry and Chemical Engineering, Southeast University, Nanjing 210096, People’s Republic of China

## Abstract

In the title compound, 2C_17_H_24_NO^+^·BF_4_^−^·F^−^·H_2_O, the asymmetric unit contains two *N*-(2-hy­droxy­benz­yl)adamantan-1-aminium cations, one BF_4_^−^ anion, one F^−^ anion and one water mol­ecule. Both amine N atoms are protonated. The hy­droxy O atoms, amino N atoms and water O atom are involved in inter­molecular O—H⋯O, O—H⋯F, N—H⋯O and N—H⋯F hydrogen bonding.

## Related literature

For the structures of related amino compounds, see: Fu *et al.* (2007 ▶, 2008 ▶, 2009 ▶); Fu & Xiong (2008 ▶). For the ferroelectric properties of related amino derivatives, see: Fu *et al.* (2011*a* ▶,*b* ▶,*c* ▶).
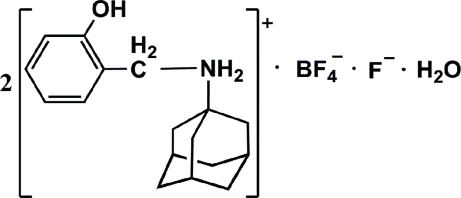


## Experimental

### 

#### Crystal data

2C_17_H_24_NO^+^·BF_4_^−^·F^−^·H_2_O
*M* *_r_* = 640.57
Triclinic, 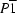

*a* = 9.4546 (19) Å
*b* = 12.532 (3) Å
*c* = 15.394 (3) Å
α = 104.28 (3)°
β = 103.20 (3)°
γ = 94.54 (3)°
*V* = 1703.1 (6) Å^3^
*Z* = 2
Mo *K*α radiation
μ = 0.10 mm^−1^
*T* = 293 K
0.10 × 0.05 × 0.05 mm

#### Data collection

Rigaku Mercury2 diffractometer
14663 measured reflections
5987 independent reflections
2943 reflections with *I* > 2σ(*I*)
*R*_int_ = 0.078

#### Refinement

*R*[*F* ^2^ > 2σ(*F* ^2^)] = 0.074
*wR*(*F* ^2^) = 0.221
*S* = 1.01
5987 reflections
406 parameters
6 restraints
H-atom parameters constrained
Δρ_max_ = 0.34 e Å^−3^
Δρ_min_ = −0.36 e Å^−3^

### 

Data collection: *CrystalClear* (Rigaku, 2005 ▶); cell refinement: *CrystalClear*; data reduction: *CrystalClear*; program(s) used to solve structure: *SHELXS97* (Sheldrick, 2008 ▶); program(s) used to refine structure: *SHELXL97* (Sheldrick, 2008 ▶); molecular graphics: *SHELXTL* (Sheldrick, 2008 ▶); software used to prepare material for publication: *SHELXTL*.

## Supplementary Material

Crystal structure: contains datablock(s) I, global. DOI: 10.1107/S1600536812000694/xu5436sup1.cif

Structure factors: contains datablock(s) I. DOI: 10.1107/S1600536812000694/xu5436Isup2.hkl

Supplementary material file. DOI: 10.1107/S1600536812000694/xu5436Isup3.cml

Additional supplementary materials:  crystallographic information; 3D view; checkCIF report

## Figures and Tables

**Table 1:** Hydrogen-bond geometry (Å, °)

*D*—H⋯*A*	*D*—H	H⋯*A*	*D*⋯*A*	*D*—H⋯*A*
N1—H1*B*⋯F3	0.90	2.26	3.125 (4)	161
N1—H1*B*⋯F4	0.90	2.26	2.992 (4)	138
N1—H1*C*⋯F5	0.90	1.74	2.607 (3)	161
N2—H2*B*⋯O2	0.90	2.13	2.791 (3)	129
N2—H2*B*⋯F3^i^	0.90	2.52	3.251 (3)	139
N2—H2*D*⋯F5^i^	0.90	1.70	2.600 (3)	175
O1—H1*A*⋯F5^ii^	0.82	1.67	2.487 (3)	172
O2—H2*A*⋯O3*W*^i^	0.82	1.85	2.660 (4)	168
O3*W*—H3*B*⋯F2^iii^	0.82	1.97	2.733 (6)	155
O3*W*—H3*C*⋯F1	0.82	2.03	2.842 (6)	169

Symmetry codes: (i) 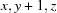; (ii) 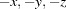; (iii) 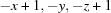.

**Fig. 1.**
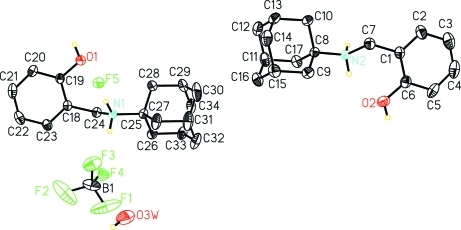
Molecular view of the title compound with the atomic numbering scheme. Displacement ellipsoids are drawn at the 30% probability level. H atoms (except the H atoms bonding to N and O atoms) have been omitted for clarity.

**Fig. 2.**
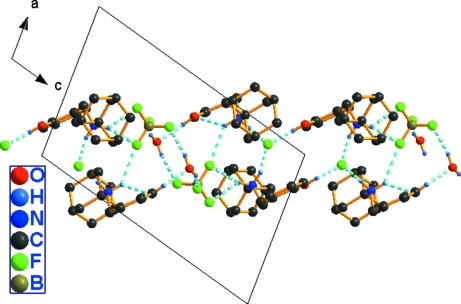
The crystal packing of the title compound viewed along the b axis showing the hydrogen bondings unit (dashed line). H atoms not involved in hydrogen bonding (dashed line) have been omitted for clarity.

## supplementary crystallographic information

### Comment

Organic amino compounds attracted more attention as phase transition dielectric materials for its application in memory storage (Fu *et al.* 2007; Fu & Xiong 2008; Fu *et al.* 2008; Fu *et al.* 2009). With the purpose of obtaining phase transition crystals of amino compounds, various amines have been studied and we have elaborated a series of new materials with this organic molecules (Fu *et al.* 2011*a*; Fu *et al.* 2011*b*; Fu *et al.* 2011*c*). In this study, we describe the crystal structure of the title compound, *bis*-N-(2-Hydroxybenzyl)adamantan-1-aminium tetrafluoroborate monofluoride monohydrate.

The asymmetric unit is composed of two N-(2-Hydroxybenzyl)adamantan-1-aminium cations, one BF_4_ anion, one F^-^ anion and one water molecule. The two benzene rings are nearly coplanar and only twisted from each other by a dihedral angle of 4.48 (2)°. Both the amine N atoms were protonated. And the BF_4_ and F^-^ groups were deprotonated to keep the charge balance. The geometric parameters of the title compound are in the normal range.

In the crystal structure, all the hydroxy O atoms, amino N atoms and aqueous O atom are involved in intermolecular O—H···O, O—H···F, N—H···O and N—H···F H-bonding interactions with the O atoms (O2 and O3W) and F atoms (F1 to F5). These hydrogen bonds link the molecules and ion units into a 1D chain parallel to the *ac*-plane(Table 1 and Fig.2).

### Experimental

A mixture of N-(2-Hydroxybenzyl)adamantan-1-amine (4.0 mmol), HBF_4_ (2.0 mL), HF (2.0 mL) and 20 mL ethanol were added into a 50ml flask and refluxed for 5 hours, then cooled and filtrated. The solution was evaporated slowly in the air. Colorless block crystals suitable for X-ray analysis were obtained after one week.

### Refinement

All H atoms attached to C atoms were fixed geometrically and treated as riding with C-H = 0.93 Å (aromatic), C-H = 0.97 Å (methylene) and C-H = 0.98 Å (methine) with *U*_iso_(H) = 1.2*U*_eq_(C). H atoms bonded to N and O atoms were located in difference Fourier maps and restrained with the H–N = 0.90 (2)Å and H—O = 0.82 (2)Å. In the last stage of refinement they were treated as riding on the N and O atoms with *U*_iso_(H) = 1.2*U*_eq_(N) and *U*_iso_(H) = 1.5*U*_eq_(O).

### Crystal data

**Table:**

2C_17_H_24_NO^+^·BF_4_^−^·F^−^·H_2_O	*Z* = 2
*M**_r_* = 640.57	*F*(000) = 684
Triclinic, *P*1	*D*_x_ = 1.249 Mg m^−^^3^
Hall symbol: -P 1	Mo *K*α radiation, λ = 0.71073 Å
*a* = 9.4546 (19) Å	Cell parameters from 5987 reflections
*b* = 12.532 (3) Å	θ = 3.0–27.5°
*c* = 15.394 (3) Å	µ = 0.10 mm^−^^1^
α = 104.28 (3)°	*T* = 293 K
β = 103.20 (3)°	Block, colorless
γ = 94.54 (3)°	0.10 × 0.05 × 0.05 mm
*V* = 1703.1 (6) Å^3^	

### Data collection

**Table:**

Rigaku Mercury2 diffractometer	2943 reflections with *I* > 2σ(*I*)
Radiation source: fine-focus sealed tube	*R*_int_ = 0.078
graphite	θ_max_ = 25.0°, θ_min_ = 3.0°
Detector resolution: 13.6612 pixels mm^-1^	*h* = −11→11
CCD profile fitting scans	*k* = −14→14
14663 measured reflections	*l* = −18→18
5987 independent reflections	

### Refinement

**Table:**

Refinement on *F*^2^	Primary atom site location: structure-invariant direct methods
Least-squares matrix: full	Secondary atom site location: difference Fourier map
*R*[*F*^2^ > 2σ(*F*^2^)] = 0.074	Hydrogen site location: inferred from neighbouring sites
*wR*(*F*^2^) = 0.221	H-atom parameters constrained
*S* = 1.00	*w* = 1/[σ^2^(*F*_o_^2^) + (0.0941*P*)^2^] where *P* = (*F*_o_^2^ + 2*F*_c_^2^)/3
5987 reflections	(Δ/σ)_max_ < 0.001
406 parameters	Δρ_max_ = 0.34 e Å^−^^3^
6 restraints	Δρ_min_ = −0.36 e Å^−^^3^

### Special details

**Table:**

Geometry. All esds (except the esd in the dihedral angle between two l.s. planes) are estimated using the full covariance matrix. The cell esds are taken into account individually in the estimation of esds in distances, angles and torsion angles; correlations between esds in cell parameters are only used when they are defined by crystal symmetry. An approximate (isotropic) treatment of cell esds is used for estimating esds involving l.s. planes.
Refinement. Refinement of F^2^ against ALL reflections. The weighted R-factor wR and goodness of fit S are based on F^2^, conventional R-factors R are based on F, with F set to zero for negative F^2^. The threshold expression of F^2^ > 2sigma(F^2^) is used only for calculating R-factors(gt) etc. and is not relevant to the choice of reflections for refinement. R-factors based on F^2^ are statistically about twice as large as those based on F, and R- factors based on ALL data will be even larger.

### Fractional atomic coordinates and isotropic or equivalent isotropic displacement parameters (Å^2^)

**Table:**

	*x*	*y*	*z*	*U*_iso_*/*U*_eq_	
O2	0.2166 (3)	1.1728 (2)	0.47603 (16)	0.0768 (7)	
H2A	0.3013	1.1960	0.5069	0.115*	
N2	0.0613 (2)	1.02915 (18)	0.30531 (17)	0.0457 (6)	
H2B	0.1500	1.0481	0.3463	0.055*	
H2D	0.0719	1.0434	0.2525	0.055*	
C1	0.0278 (4)	1.2233 (3)	0.3748 (2)	0.0559 (9)	
C2	−0.0385 (4)	1.3028 (3)	0.3406 (3)	0.0823 (12)	
H2C	−0.1256	1.2820	0.2940	0.099*	
C3	0.0221 (6)	1.4134 (4)	0.3743 (4)	0.1035 (16)	
H3A	−0.0252	1.4669	0.3515	0.124*	
C4	0.1489 (7)	1.4435 (4)	0.4398 (4)	0.0999 (16)	
H4A	0.1894	1.5182	0.4617	0.120*	
C5	0.2210 (5)	1.3663 (4)	0.4756 (3)	0.0836 (13)	
H5A	0.3099	1.3878	0.5206	0.100*	
C6	0.1572 (4)	1.2556 (3)	0.4427 (3)	0.0623 (9)	
C7	−0.0398 (4)	1.1038 (3)	0.3424 (3)	0.0611 (9)	
H7A	−0.1296	1.0952	0.2942	0.073*	
H7B	−0.0653	1.0817	0.3936	0.073*	
C8	0.0186 (3)	0.9063 (2)	0.2855 (2)	0.0442 (7)	
C9	0.1294 (4)	0.8504 (3)	0.2392 (3)	0.0616 (9)	
H9A	0.2274	0.8747	0.2800	0.074*	
H9B	0.1273	0.8713	0.1823	0.074*	
C10	−0.1343 (3)	0.8703 (3)	0.2206 (2)	0.0597 (9)	
H10A	−0.1380	0.8915	0.1637	0.072*	
H10B	−0.2056	0.9060	0.2496	0.072*	
C11	0.0925 (4)	0.7243 (3)	0.2173 (3)	0.0713 (11)	
H11A	0.1639	0.6881	0.1875	0.086*	
C12	−0.0605 (5)	0.6881 (3)	0.1526 (3)	0.0790 (12)	
H12A	−0.0637	0.7090	0.0957	0.095*	
H12B	−0.0848	0.6079	0.1371	0.095*	
C13	−0.1698 (4)	0.7428 (3)	0.1991 (3)	0.0727 (11)	
H13A	−0.2685	0.7181	0.1575	0.087*	
C14	0.0981 (4)	0.6935 (3)	0.3076 (3)	0.0766 (11)	
H14A	0.0764	0.6134	0.2950	0.092*	
H14B	0.1960	0.7174	0.3487	0.092*	
C15	−0.1649 (4)	0.7114 (3)	0.2891 (3)	0.0807 (12)	
H15A	−0.1909	0.6316	0.2759	0.097*	
H15B	−0.2353	0.7475	0.3188	0.097*	
C16	−0.0107 (4)	0.7479 (3)	0.3536 (3)	0.0708 (11)	
H16A	−0.0072	0.7268	0.4112	0.085*	
C17	0.0250 (4)	0.8748 (3)	0.3757 (2)	0.0614 (9)	
H17A	−0.0454	0.9102	0.4059	0.074*	
H17B	0.1222	0.8999	0.4174	0.074*	
O1	0.1598 (2)	−0.01917 (18)	−0.04354 (16)	0.0671 (7)	
H1A	0.0796	−0.0455	−0.0799	0.101*	
N1	0.3591 (3)	0.12722 (18)	0.15517 (16)	0.0458 (6)	
H1B	0.3973	0.1208	0.2125	0.055*	
H1C	0.2626	0.1027	0.1428	0.055*	
C18	0.3497 (3)	−0.0654 (2)	0.0606 (2)	0.0474 (8)	
C19	0.2181 (4)	−0.0990 (2)	−0.0076 (2)	0.0493 (8)	
C20	0.1529 (4)	−0.2079 (3)	−0.0355 (2)	0.0622 (9)	
H20A	0.0652	−0.2300	−0.0816	0.075*	
C21	0.2177 (5)	−0.2837 (3)	0.0049 (3)	0.0766 (12)	
H21A	0.1726	−0.3573	−0.0135	0.092*	
C22	0.3476 (5)	−0.2532 (3)	0.0719 (3)	0.0759 (12)	
H22A	0.3912	−0.3054	0.0986	0.091*	
C23	0.4205 (3)	0.0525 (2)	0.0859 (2)	0.0514 (8)	
H23A	0.4063	0.0782	0.0304	0.062*	
H23B	0.5252	0.0565	0.1112	0.062*	
C24	0.3909 (3)	0.2505 (2)	0.1669 (2)	0.0470 (8)	
C25	0.4134 (4)	−0.1434 (3)	0.0993 (2)	0.0592 (9)	
H25A	0.5019	−0.1221	0.1447	0.071*	
C26	0.5540 (4)	0.2863 (3)	0.1827 (3)	0.0670 (10)	
H26A	0.5866	0.2486	0.1293	0.080*	
H26B	0.6087	0.2668	0.2366	0.080*	
C27	0.5349 (6)	0.4692 (3)	0.2807 (3)	0.1109 (17)	
H27A	0.5549	0.5491	0.2911	0.133*	
H27B	0.5902	0.4504	0.3349	0.133*	
C28	0.5812 (4)	0.4116 (3)	0.1977 (3)	0.0825 (12)	
H28A	0.6863	0.4351	0.2073	0.099*	
C29	0.4980 (5)	0.4424 (3)	0.1150 (3)	0.0837 (13)	
H29A	0.5307	0.4069	0.0610	0.100*	
H29B	0.5165	0.5223	0.1250	0.100*	
C30	0.2869 (6)	0.4637 (3)	0.1814 (5)	0.124 (2)	
H30A	0.1828	0.4406	0.1716	0.149*	
H30B	0.3028	0.5436	0.1906	0.149*	
C31	0.3359 (5)	0.4061 (3)	0.0979 (3)	0.0877 (13)	
H31A	0.2821	0.4262	0.0432	0.105*	
C32	0.3073 (4)	0.2796 (3)	0.0817 (3)	0.0702 (11)	
H32A	0.2031	0.2554	0.0702	0.084*	
H32B	0.3393	0.2423	0.0280	0.084*	
C33	0.3429 (5)	0.3085 (3)	0.2515 (3)	0.0847 (13)	
H33A	0.3965	0.2885	0.3055	0.102*	
H33B	0.2389	0.2856	0.2425	0.102*	
C34	0.3730 (7)	0.4347 (3)	0.2667 (4)	0.114 (2)	
H34A	0.3422	0.4732	0.3214	0.136*	
F1	0.6340 (4)	0.1272 (5)	0.4430 (3)	0.258 (3)	
F2	0.5834 (7)	−0.0399 (5)	0.3596 (4)	0.286 (4)	
F3	0.4169 (3)	0.0710 (3)	0.3459 (2)	0.1287 (10)	
F4	0.6105 (3)	0.0848 (3)	0.2942 (2)	0.1330 (11)	
B1	0.5602 (7)	0.0654 (7)	0.3648 (5)	0.112 (2)	
O3W	0.4910 (3)	0.2203 (3)	0.5815 (2)	0.1357 (13)	
H3C	0.5421	0.1997	0.5458	0.204*	
H3B	0.4798	0.1802	0.6150	0.204*	
F5	0.08538 (18)	0.08174 (14)	0.15565 (12)	0.0593 (5)	

### Atomic displacement parameters (Å^2^)

**Table:**

	*U*^11^	*U*^22^	*U*^33^	*U*^12^	*U*^13^	*U*^23^
O2	0.0752 (17)	0.0827 (18)	0.0604 (16)	0.0041 (14)	0.0026 (13)	0.0132 (14)
N2	0.0396 (14)	0.0525 (16)	0.0467 (16)	0.0023 (12)	0.0168 (12)	0.0129 (13)
C1	0.061 (2)	0.051 (2)	0.062 (2)	0.0100 (18)	0.0286 (19)	0.0148 (18)
C2	0.076 (3)	0.064 (3)	0.115 (4)	0.021 (2)	0.036 (3)	0.027 (3)
C3	0.121 (4)	0.070 (3)	0.144 (5)	0.026 (3)	0.061 (4)	0.043 (3)
C4	0.130 (5)	0.054 (3)	0.125 (5)	0.001 (3)	0.070 (4)	0.009 (3)
C5	0.100 (3)	0.070 (3)	0.065 (3)	−0.015 (3)	0.030 (2)	−0.011 (2)
C6	0.074 (3)	0.061 (2)	0.053 (2)	0.010 (2)	0.027 (2)	0.008 (2)
C7	0.050 (2)	0.060 (2)	0.077 (2)	0.0087 (17)	0.0245 (18)	0.0161 (19)
C8	0.0399 (17)	0.0446 (18)	0.0499 (19)	0.0008 (14)	0.0158 (15)	0.0139 (15)
C9	0.064 (2)	0.059 (2)	0.077 (3)	0.0148 (17)	0.036 (2)	0.0271 (19)
C10	0.049 (2)	0.057 (2)	0.065 (2)	−0.0002 (16)	0.0062 (17)	0.0116 (18)
C11	0.088 (3)	0.056 (2)	0.089 (3)	0.021 (2)	0.051 (3)	0.025 (2)
C12	0.113 (4)	0.051 (2)	0.067 (3)	0.000 (2)	0.025 (3)	0.008 (2)
C13	0.059 (2)	0.063 (2)	0.079 (3)	−0.0082 (18)	0.004 (2)	0.007 (2)
C14	0.084 (3)	0.062 (2)	0.098 (3)	0.017 (2)	0.033 (2)	0.037 (2)
C15	0.085 (3)	0.058 (2)	0.111 (4)	−0.003 (2)	0.051 (3)	0.025 (2)
C16	0.089 (3)	0.071 (2)	0.070 (3)	0.008 (2)	0.033 (2)	0.039 (2)
C17	0.068 (2)	0.068 (2)	0.051 (2)	−0.0004 (18)	0.0171 (18)	0.0224 (18)
O1	0.0626 (15)	0.0603 (14)	0.0734 (17)	0.0008 (12)	0.0021 (13)	0.0258 (13)
N1	0.0434 (14)	0.0490 (15)	0.0448 (15)	0.0015 (12)	0.0140 (12)	0.0112 (12)
C18	0.051 (2)	0.0438 (18)	0.053 (2)	0.0091 (15)	0.0253 (17)	0.0122 (16)
C19	0.056 (2)	0.0450 (19)	0.050 (2)	0.0055 (16)	0.0205 (17)	0.0141 (17)
C20	0.063 (2)	0.054 (2)	0.066 (2)	−0.0025 (18)	0.0250 (19)	0.0059 (19)
C21	0.098 (3)	0.045 (2)	0.100 (3)	0.009 (2)	0.059 (3)	0.014 (2)
C22	0.092 (3)	0.063 (3)	0.098 (3)	0.034 (2)	0.050 (3)	0.039 (2)
C23	0.0465 (19)	0.0520 (19)	0.059 (2)	0.0082 (15)	0.0235 (17)	0.0115 (17)
C24	0.0460 (18)	0.0439 (18)	0.050 (2)	0.0024 (14)	0.0181 (15)	0.0067 (15)
C25	0.061 (2)	0.055 (2)	0.071 (2)	0.0196 (18)	0.0279 (19)	0.0212 (19)
C26	0.050 (2)	0.062 (2)	0.084 (3)	−0.0023 (17)	0.0122 (19)	0.018 (2)
C27	0.151 (5)	0.062 (3)	0.100 (4)	−0.032 (3)	0.039 (4)	−0.006 (3)
C28	0.069 (3)	0.058 (2)	0.110 (4)	−0.0161 (19)	0.026 (3)	0.009 (3)
C29	0.110 (4)	0.049 (2)	0.110 (4)	0.010 (2)	0.059 (3)	0.026 (2)
C30	0.115 (4)	0.051 (3)	0.228 (7)	0.025 (3)	0.096 (5)	0.027 (4)
C31	0.104 (3)	0.058 (2)	0.108 (4)	0.026 (2)	0.024 (3)	0.033 (2)
C32	0.066 (2)	0.056 (2)	0.084 (3)	0.0081 (18)	0.005 (2)	0.025 (2)
C33	0.122 (4)	0.054 (2)	0.086 (3)	0.000 (2)	0.065 (3)	0.000 (2)
C34	0.182 (6)	0.058 (3)	0.122 (4)	0.006 (3)	0.107 (4)	0.002 (3)
F1	0.120 (3)	0.491 (9)	0.084 (2)	−0.005 (4)	−0.011 (2)	−0.014 (4)
F2	0.337 (7)	0.333 (7)	0.349 (8)	0.201 (6)	0.168 (7)	0.262 (7)
F3	0.0670 (17)	0.177 (3)	0.129 (2)	0.0101 (17)	0.0298 (16)	0.017 (2)
F4	0.0848 (18)	0.216 (3)	0.121 (2)	0.0286 (19)	0.0281 (17)	0.083 (2)
B1	0.079 (4)	0.179 (7)	0.095 (5)	0.044 (4)	0.020 (4)	0.066 (5)
O3W	0.094 (2)	0.161 (3)	0.121 (3)	0.010 (2)	−0.001 (2)	0.013 (2)
F5	0.0519 (11)	0.0727 (12)	0.0573 (12)	0.0026 (9)	0.0173 (9)	0.0241 (10)

### Geometric parameters (Å, °)

**Table:**

O2—C6	1.370 (4)	N1—C24	1.508 (3)
O2—H2A	0.8200	N1—H1B	0.9001
N2—C8	1.496 (3)	N1—H1C	0.9001
N2—C7	1.498 (4)	C18—C25	1.375 (4)
N2—H2B	0.9000	C18—C19	1.389 (4)
N2—H2D	0.9000	C18—C23	1.491 (4)
C1—C2	1.368 (5)	C19—C20	1.372 (4)
C1—C6	1.372 (5)	C20—C21	1.370 (5)
C1—C7	1.495 (4)	C20—H20A	0.9300
C2—C3	1.379 (5)	C21—C22	1.368 (5)
C2—H2C	0.9300	C21—H21A	0.9300
C3—C4	1.336 (7)	C22—C25	1.384 (5)
C3—H3A	0.9300	C22—H22A	0.9300
C4—C5	1.379 (6)	C23—H23A	0.9700
C4—H4A	0.9300	C23—H23B	0.9700
C5—C6	1.388 (5)	C24—C33	1.512 (4)
C5—H5A	0.9300	C24—C32	1.512 (4)
C7—H7A	0.9700	C24—C26	1.518 (4)
C7—H7B	0.9700	C25—H25A	0.9300
C8—C10	1.519 (4)	C26—C28	1.522 (4)
C8—C9	1.520 (4)	C26—H26A	0.9700
C8—C17	1.525 (4)	C26—H26B	0.9700
C9—C11	1.526 (4)	C27—C28	1.483 (5)
C9—H9A	0.9700	C27—C34	1.509 (7)
C9—H9B	0.9700	C27—H27A	0.9700
C10—C13	1.543 (4)	C27—H27B	0.9700
C10—H10A	0.9700	C28—C29	1.490 (5)
C10—H10B	0.9700	C28—H28A	0.9800
C11—C12	1.518 (5)	C29—C31	1.506 (5)
C11—C14	1.522 (5)	C29—H29A	0.9700
C11—H11A	0.9800	C29—H29B	0.9700
C12—C13	1.502 (5)	C30—C31	1.502 (6)
C12—H12A	0.9700	C30—C34	1.522 (7)
C12—H12B	0.9700	C30—H30A	0.9700
C13—C15	1.523 (5)	C30—H30B	0.9700
C13—H13A	0.9800	C31—C32	1.534 (5)
C14—C16	1.494 (5)	C31—H31A	0.9800
C14—H14A	0.9700	C32—H32A	0.9700
C14—H14B	0.9700	C32—H32B	0.9700
C15—C16	1.527 (5)	C33—C34	1.533 (5)
C15—H15A	0.9700	C33—H33A	0.9700
C15—H15B	0.9700	C33—H33B	0.9700
C16—C17	1.534 (4)	C34—H34A	0.9800
C16—H16A	0.9800	F1—B1	1.268 (8)
C17—H17A	0.9700	F2—B1	1.341 (8)
C17—H17B	0.9700	F3—B1	1.331 (6)
O1—C19	1.354 (3)	F4—B1	1.347 (6)
O1—H1A	0.8200	O3W—H3C	0.8214
N1—C23	1.492 (3)	O3W—H3B	0.8203
			
C6—O2—H2A	109.5	C23—N1—H1C	107.9
C8—N2—C7	117.7 (2)	C24—N1—H1C	112.9
C8—N2—H2B	107.9	H1B—N1—H1C	104.9
C7—N2—H2B	107.9	C25—C18—C19	118.7 (3)
C8—N2—H2D	107.9	C25—C18—C23	122.4 (3)
C7—N2—H2D	107.9	C19—C18—C23	118.9 (3)
H2B—N2—H2D	107.2	O1—C19—C20	122.9 (3)
C2—C1—C6	118.6 (3)	O1—C19—C18	116.5 (3)
C2—C1—C7	121.6 (4)	C20—C19—C18	120.6 (3)
C6—C1—C7	119.8 (3)	C21—C20—C19	119.6 (4)
C1—C2—C3	120.8 (4)	C21—C20—H20A	120.2
C1—C2—H2C	119.6	C19—C20—H20A	120.2
C3—C2—H2C	119.6	C22—C21—C20	121.1 (3)
C4—C3—C2	119.9 (5)	C22—C21—H21A	119.4
C4—C3—H3A	120.1	C20—C21—H21A	119.4
C2—C3—H3A	120.1	C21—C22—C25	119.0 (4)
C3—C4—C5	121.4 (4)	C21—C22—H22A	120.5
C3—C4—H4A	119.3	C25—C22—H22A	120.5
C5—C4—H4A	119.3	C18—C23—N1	112.1 (2)
C4—C5—C6	118.2 (4)	C18—C23—H23A	109.2
C4—C5—H5A	120.9	N1—C23—H23A	109.2
C6—C5—H5A	120.9	C18—C23—H23B	109.2
O2—C6—C1	116.0 (3)	N1—C23—H23B	109.2
O2—C6—C5	123.0 (4)	H23A—C23—H23B	107.9
C1—C6—C5	121.0 (4)	N1—C24—C33	106.8 (2)
C1—C7—N2	112.0 (2)	N1—C24—C32	110.1 (3)
C1—C7—H7A	109.2	C33—C24—C32	110.7 (3)
N2—C7—H7A	109.2	N1—C24—C26	110.7 (2)
C1—C7—H7B	109.2	C33—C24—C26	109.1 (3)
N2—C7—H7B	109.2	C32—C24—C26	109.4 (3)
H7A—C7—H7B	107.9	C18—C25—C22	121.0 (4)
N2—C8—C10	110.3 (2)	C18—C25—H25A	119.5
N2—C8—C9	106.9 (2)	C22—C25—H25A	119.5
C10—C8—C9	109.7 (3)	C24—C26—C28	108.7 (3)
N2—C8—C17	110.1 (2)	C24—C26—H26A	109.9
C10—C8—C17	110.5 (2)	C28—C26—H26A	109.9
C9—C8—C17	109.3 (3)	C24—C26—H26B	109.9
C8—C9—C11	109.7 (2)	C28—C26—H26B	109.9
C8—C9—H9A	109.7	H26A—C26—H26B	108.3
C11—C9—H9A	109.7	C28—C27—C34	109.7 (4)
C8—C9—H9B	109.7	C28—C27—H27A	109.7
C11—C9—H9B	109.7	C34—C27—H27A	109.7
H9A—C9—H9B	108.2	C28—C27—H27B	109.7
C8—C10—C13	108.0 (3)	C34—C27—H27B	109.7
C8—C10—H10A	110.1	H27A—C27—H27B	108.2
C13—C10—H10A	110.1	C27—C28—C29	109.5 (4)
C8—C10—H10B	110.1	C27—C28—C26	109.9 (3)
C13—C10—H10B	110.1	C29—C28—C26	110.3 (3)
H10A—C10—H10B	108.4	C27—C28—H28A	109.0
C12—C11—C14	110.2 (3)	C29—C28—H28A	109.0
C12—C11—C9	108.8 (3)	C26—C28—H28A	109.0
C14—C11—C9	108.7 (3)	C28—C29—C31	110.2 (3)
C12—C11—H11A	109.7	C28—C29—H29A	109.6
C14—C11—H11A	109.7	C31—C29—H29A	109.6
C9—C11—H11A	109.7	C28—C29—H29B	109.6
C13—C12—C11	109.5 (3)	C31—C29—H29B	109.6
C13—C12—H12A	109.8	H29A—C29—H29B	108.1
C11—C12—H12A	109.8	C31—C30—C34	109.7 (4)
C13—C12—H12B	109.8	C31—C30—H30A	109.7
C11—C12—H12B	109.8	C34—C30—H30A	109.7
H12A—C12—H12B	108.2	C31—C30—H30B	109.7
C12—C13—C15	110.3 (3)	C34—C30—H30B	109.7
C12—C13—C10	109.8 (3)	H30A—C30—H30B	108.2
C15—C13—C10	109.2 (3)	C30—C31—C29	108.8 (4)
C12—C13—H13A	109.2	C30—C31—C32	109.8 (3)
C15—C13—H13A	109.2	C29—C31—C32	109.4 (3)
C10—C13—H13A	109.2	C30—C31—H31A	109.6
C16—C14—C11	110.2 (3)	C29—C31—H31A	109.6
C16—C14—H14A	109.6	C32—C31—H31A	109.6
C11—C14—H14A	109.6	C24—C32—C31	108.7 (3)
C16—C14—H14B	109.6	C24—C32—H32A	109.9
C11—C14—H14B	109.6	C31—C32—H32A	109.9
H14A—C14—H14B	108.1	C24—C32—H32B	109.9
C13—C15—C16	109.5 (3)	C31—C32—H32B	109.9
C13—C15—H15A	109.8	H32A—C32—H32B	108.3
C16—C15—H15A	109.8	C24—C33—C34	109.0 (3)
C13—C15—H15B	109.8	C24—C33—H33A	109.9
C16—C15—H15B	109.8	C34—C33—H33A	109.9
H15A—C15—H15B	108.2	C24—C33—H33B	109.9
C14—C16—C15	109.9 (3)	C34—C33—H33B	109.9
C14—C16—C17	109.9 (3)	H33A—C33—H33B	108.3
C15—C16—C17	108.5 (3)	C27—C34—C30	109.5 (4)
C14—C16—H16A	109.5	C27—C34—C33	109.3 (4)
C15—C16—H16A	109.5	C30—C34—C33	108.7 (4)
C17—C16—H16A	109.5	C27—C34—H34A	109.8
C8—C17—C16	108.9 (3)	C30—C34—H34A	109.8
C8—C17—H17A	109.9	C33—C34—H34A	109.8
C16—C17—H17A	109.9	F1—B1—F3	114.8 (6)
C8—C17—H17B	109.9	F1—B1—F2	108.8 (6)
C16—C17—H17B	109.9	F3—B1—F2	110.1 (7)
H17A—C17—H17B	108.3	F1—B1—F4	112.5 (7)
C19—O1—H1A	109.5	F3—B1—F4	108.1 (5)
C23—N1—C24	116.8 (2)	F2—B1—F4	101.8 (6)
C23—N1—H1B	110.6	H3C—O3W—H3B	115.9
C24—N1—H1B	103.1		

### Hydrogen-bond geometry (Å, °)

**Table:**

*D*—H···*A*	*D*—H	H···*A*	*D*···*A*	*D*—H···*A*
N1—H1B···F3	0.90	2.26	3.125 (4)	161
N1—H1B···F4	0.90	2.26	2.992 (4)	138
N1—H1C···F5	0.90	1.74	2.607 (3)	161
N2—H2B···O2	0.90	2.13	2.791 (3)	129
N2—H2B···F3^i^	0.90	2.52	3.251 (3)	139
N2—H2D···F5^i^	0.90	1.70	2.600 (3)	175
O1—H1A···F5^ii^	0.82	1.67	2.487 (3)	172
O2—H2A···O3W^i^	0.82	1.85	2.660 (4)	168
O3W—H3B···F2^iii^	0.82	1.97	2.733 (6)	155
O3W—H3C···F1	0.82	2.03	2.842 (6)	169

Symmetry codes: (i) *x*, *y*+1, *z*; (ii) −*x*, −*y*, −*z*; (iii) −*x*+1, −*y*, −*z*+1.
